# If the party is good, you can stay longer—effects of trait hedonic capacity on hedonic quantity and performance

**DOI:** 10.1007/s11031-023-10021-6

**Published:** 2023-05-22

**Authors:** Katharina Bernecker, Daniela Becker, Aiste Guobyte

**Affiliations:** 1 University of Zurich, Zurich, Switzerland; 2 URPP “Dynamics of Healthy Aging”, University of Zurich, Zurich, Switzerland; 3 Radboud University, Nijmegen, The Netherlands

**Keywords:** Hedonic goals, Performance, Self-control, Goal pursuit

## Abstract

Research suggests that people’s capacity to successfully pursue hedonic goals is at least as important for well-being as trait self-control. Extending this research, we tested whether trait hedonic capacity is related to more time spent with hedonic goal pursuit (i.e., hedonic quantity) and whether this explains its positive relationship with well-being. Second, we explored whether this may come at a cost for people’s performance. Results show that people with higher trait hedonic capacity do spend more time with hedonic goal pursuit (Study 1 and 2). However, hedonic quality not hedonic quantity accounts for its positive relationship with well-being. Further, people higher vs. lower in trait hedonic capacity perform equally well in their studies (Study 2) and their jobs (Study 3 and 4). Thus, trait hedonic capacity seems to allow people to invest more time into their hedonic goals in a way that does not jeopardize their academic and job performance.

In the past decades, research on self-control has documented the positive effects of people’s trait self-control, that is their capacity to forego short-term desires in the service of their long-term goals and values (Baumeister & Heatherton, 1996; Kotabe & Hofmann, 2015). Among other things, people with higher levels of trait self-control experience greater well-being and psychological adjustment and perform better on their studies and their jobs (de Ridder et al., 2012; Moffitt et al., 2011). It is perhaps because of this adaptive function that the self-control literature has demonized people’s short-term motivations, such as desires or impulses, as potential threats to people’s long-term endeavors and conceptualized their suppression as part of adaptive self-regulation.

Challenging this view, recent research suggests that successful pursuit of hedonic goals is at least equally important for well-being and that it poses its own self-regulatory problem (Bernecker & Becker, 2021). That is, people sometimes seek immediate pleasure (i.e., pursue a hedonic goal) but they do not always get to experience the positive affective states they are looking for. One reason for that is that people can get distracted by intrusive thoughts about conflicting long-term goals or values. Similar to trait self-control, people also differ in their capacity to pursue hedonic goals. Studies showed that people who are more successful not only experience a higher quality of their hedonic experiences in everyday life but also report substantially greater subjective well-being (Bernecker & Becker, 2021). Thus, at least in terms of people’s well-being, the successful pursuit of short-term pleasures seems to be adaptive rather than maladaptive. Nonetheless, the self-control literature might be right in proposing that engaging in hedonic goal pursuit too often may pose a threat to people’s long-term goals. Therefore, the present research extends previous research in several ways by, first, testing whether trait hedonic capacity is related to spending more (or less) time with hedonic goal pursuit in everyday life and whether this might explain its positive effects on well-being. Second, we explored whether there might be a potential downside of higher trait hedonic capacity for people’s academic or job performance.

## Relevance of trait self-control and trait hedonic capacity for well-being

With regard to well-being, quite a large number of studies document positive relationships between trait self-control and people’s so called cognitive (e.g., life satisfaction) and affective well-being (e.g., positive affect; Diener et al., 1999). A meta-analysis conducted in 2012, found a medium-sized effect (*r* = .33, k = 16, *N* = 4946), that was relatively heterogeneous, which may be explained by the fact that indicators of well-being were grouped together with indicators of psychological adjustment (de Ridder et al., 2012). More recent research suggests that the relationship between self-control and well-being seems to be linear rather than u-shaped, meaning that there is no indication that too much self-control is detrimental to well-being (Wiese et al., 2018).

Studies have also examined *why* self-control contributes to well-being and discovered a range of different mechanisms. For example, avoidance of motivational conflicts (Hofmann et al., 2014), more promotion focus and less prevention focus (Cheung et al., 2014), initiation of desired behavior and adaptive routines (de Ridder & Gillebaart, 2017), and making progress on long-term goals (Bernecker, Herrmann, Brandstätter, & Job, 2017). The current consensus of this research is that people higher in trait self-control are not necessarily more successful at suppressing short-term motivations (see also Imhoff et al., 2013), but rather, they are more successful at avoiding situations that may lead to motivational conflict, for instance, by employing strategies or building adaptive routines (de Ridder & Gillebaart, 2017; Galla & Duckworth, 2015; Hennecke et al., 2019).

In line with the idea that successful self-control is *not* about the suppression of short-term motivations, the concept of *hedonic goal pursuit* was introduced, which describes the intended rather than unwanted pursuit of short-term pleasure (Bernecker & Becker, 2021; see Hofmann & Van Dillen 2012; Inzlicht & Schmeichel, 2012 for similar ideas). However,  even though people sometimes intentionally seek short-term pleasure they also encounter motivational conflict in these situations. More specifically, they get distracted by conflicting long-term goals  (e.g., people may fail to enjoy the chocolate cake because of thoughts about their conflicting dieting goal). This is because people generally pursue multiple goals and are motivated to maximize outcomes in the present and the future which oftentimes requires different means but the same limited resources (Kruglanski et al., 2002; Mees & Schmitt, 2008).

2002) Accordingly, we found that hedonic goal pursuit can be undermined by intrusive thoughts about conflicting long-term goals (Bernecker & Becker, 2021). Further, some people are more prone to experience intrusive thoughts than others. Those differences in *trait hedonic capacity* were positively related to different indicators of well-being, such as positive affect, life satisfaction, and the absence of physical symptoms of somatization, depression, and anxiety. The effects were medium-to-large in size and independent of the effects of trait self-control on well-being (Bernecker & Becker, 2021). These findings mirror theoretical considerations and empirical work on people’s orientation towards happiness, which suggest that people can achieve well-being through an eudaimonic (i.e., engagement, meaning) and/or hedonic (i.e., pleasure) route (e.g., Peterson et al., 2005; Schueller & Seligman, 2010). The mechanisms by which these orientations work are, however, not yet well understood.

Given that the pursuit of hedonic goals is an important yet neglected part of self-regulation, the present research aimed to gain a better understanding of *why* trait hedonic capacity is positively linked to well-being. More specifically, we examined whether trait hedonic capacity is not only related to quality but also to quantity of hedonic goal pursuit and test both as possible mediators for its positive relationship with well-being.

## Relevance of trait self-control and trait hedonic capacity for academic and job performance

Studies suggest that trait self-control contributes to academic performance. For instance, Tangney and colleagues (2004), found that students with higher self-control reported better grades than those reporting low self-control, even when controlling for social desirability (Tangney et al., 2004). In another study, Moffitt and colleagues (2011) compared same-gender dizygotic siblings and found that the sibling with poorer self-control at the age of 5 was significantly more likely to perform worse at school (rated by their teachers) at the age of 12 (Moffitt et al., 2011). A more recent study, investigated effects of trait self-control on academic performance in three cohorts of engineering students and found that self-control was positively related to student’s grade point average (Honken et al., 2016).

In comparison to academic performance, less is known about the relationship between self-control and work performance. However, there is some evidence that it is positive as well. For instance, Cox (2000) found that leaders with higher self-control received more favorable performance ratings from their subordinates (as cited in Baumeister & Alquist, 2009). Another study differentiated between two aspects of self-control and found that initiatory self-control was positively related to organizational citizenship behavior and employee engagement, and inhibitory self-control negatively to counterproductive work behavior (De Boer et al., 2015). Other studies looked specifically at income as one indicator of occupational performance and found small positive correlations with self-control (Converse et al., 2012, 2014). In terms of effect size, the previously mentioned meta-analysis found a medium to strong positive effect of trait self-control on school and work performance (*r* = .36, k = 5, *N* = 1546). However, the number of studies was relatively small.

Compared to well-being, knowledge about mediating mechanisms for the self-control-performance link is relatively scarce. Taking a developmental perspective, Converse and colleagues (Converse et al., 2014) found that childhood self-control predicted positive and negative behaviors during adolescence (e.g., more studying, less drug use), which in turn predicted participants levels of education and career success as young adults (Converse et al., 2014, for similar findings see Converse et al., 2012). Focusing on smartphone use as one specific behavior that potentially undermines academic performance, recent research suggest that it is not the amount of smartphone-use (i.e., screen time) but the effective handling of smartphones that helps students with higher trait self-control to perform better (Troll et al., 2021). More specifically, students with higher trait self-control had better smartphone habits (e.g., turning sound off) and engaged less in procrastination using their phones (Troll et al., 2021). These findings suggest that it might not be the quantity of hedonic behaviors that undermines performance but rather how hedonic goals are managed, for instance, whether they are pursued in a situation when long-term goals should be prioritized. In a similar vein, Jia, Hirt, and Koh (2019) found that more successful college students do not indulge in college sports events less often but are more strategic about it (Jia et al., 2019). According to their findings, high GPA students are more sensitive to good opportunities when to go watch a game and planned compensatory studying on non-game days.

Together these findings suggest that even if trait hedonic capacity is positively related to hedonic quantity, this may not necessarily come at a cost to people’s academic or job performance.

## Trait hedonic capacity and hedonic quality and quantity

But then how is trait hedonic capacity related to how successful people pursue hedonic goals and how often they do so? In previous research, experience-sampling and field studies showed that trait hedonic capacity is positively related to the quality of hedonic goal pursuit in everyday life. More specifically, trait hedonic capacity was positively related to enjoyment of people’s hedonic activities when being approached in a park, on a hike, in a café, or after doing yoga (Bernecker & Becker, 2021, Study 3). Similarly, levels of enjoyment, positive affect, and relaxation were higher during various hedonic activities participants engaged in over the course of a 7-day experience-sampling phase (Bernecker & Becker, 2021, Study 4).

Previous research did not address how trait hedonic capacity is related to hedonic quantity, that is, whether people with higher trait hedonic capacity engage in hedonic goal pursuit more or less often. It is possible that people with higher trait hedonic capacity spent more time pursuing hedonic goals, because they initiate these activities more often and/or are persisting in these activities longer. Indeed, from a reinforcement perspective, higher quality of hedonic goal pursuit should be positively related to persistence and repetition of hedonic goal pursuit (Woolley & Fishbach, 2016). However, according to the hedonic principle, quality of hedonic goal pursuit might also be negatively related to hedonic quantity: If people manage to effectively increase their positive affect by engaging in a hedonic activities they should be more likely to start investing again into their longer term well-being (Taquet et al., 2016). Due to these competing theoretical arguments, we decided to first explore the relationship between trait hedonic capacity and hedonic quantity. Further, if it was related to hedonic quantity, we aimed to test whether engaging in hedonic goal pursuit more or less often can account for the positive relationship between trait hedonic capacity and well-being.

## The present research

The present research extends previous research in two ways: First, we explored how trait hedonic capacity is related to hedonic quantity and tested hedonic quantity (vs. hedonic quality) as mediator for its positive relationship with well-being. Second, we examined whether higher trait hedonic capacity might come at a cost to people’s academic or job performance. Study 1 and 2 target the first research question and establish the positive relationship between trait hedonic capacity and hedonic quantity and test its possible role as mediator. Study 2 additionally explores the link between trait hedonic capacity and academic performance. Study 3 and 4 test the link between trait hedonic capacity and job performance in three diverse adult samples.

All study materials, data, and code are publicly available on the Open Science Framework: https://osf.io/fvjez/?view_only=b57f25deb1dd4d2db019f54f8b4667b4. In all studies, we aimed to recruit at least *N* = 160 participants, because correlations stabilize at this sample size (Schönbrodt & Perugini, 2013) and it provides sufficient power to detect a small effect size in a multiple regression analysis, 1 – β = 0.87, *f*^2^ = 0.05, α = 0.05.

## Study 1

Aim of this study was to explore whether trait hedonic capacity is related to the time people spend with hedonic activities (i.e., hedonic quantity) and whether this might account for the relationship between trait hedonic capacity and well-being.

## Method

### Sample and procedure

We recruited a community sample of *N* = 301 adults (246 female, 51 male, 2 diverse, *M*_Age_ = 27.77 years, *SD* = 10.90, Range: 18 to 74) during lockdown due to the COVID-19 pandemic. The recruitment was carried out mainly online (e.g., Facebook, Linked-In) and in different leisure clubs and associations in the city of Zurich. With this sample size we had a power of 1 – β = 0.98 to find a small-sized effect in a multiple regression analysis (*f*^2^ = 0.05, α = 0.05).

Participants completed an 8-min online survey on “leisure activities during lockdown” in March/April 2021. Participants could win one out of two 100 CHF vouchers for a website offering outdoor activities. After providing demographics participants reported on their trait hedonic capacity and time spent with hedonic activities. Measures were administered in the order as presented below.

### Measures

**Well-being.** We assessed well-being with the WHO-5 general well-being scale (Brähler et al., 2007), which consists of five items (e.g., “I have felt cheerful and in good spirits”) rated on a scale from 1 = *never* to 6 = *all the time*. Further, we administered the Brief Symptom Inventory (Franke et al., 2017) consisting of 18 symptoms reflecting somatization (e.g., “Faintness or dizziness”), depression (e.g., “Feeling no interest in things”), and anxiety (e.g., “Nervousness or shakiness inside”), rated on a 5-point scale from 1 = *not at all* to 5 = *nearly every day*. Because the results were consistent across symptom categories and reliability was high for all 18 symptoms (Cronbach’s α = 0.86), results are reported for the averaged indicator.

**Hedonic quantity.** First, we instructed participants that we were interested in time they spend pursuing hedonic activities, which were described to them as activities that provide them with positive feelings (e.g., pleasure, enjoyment, fun). Next, participants indicated on two items how many hours they spent per day with such activities within the past two weeks on (1) a typical workday and (2) on a typical weekend day (Sat or Sun). They responded on an 11-point scale from 0 = < *1 h* to 10 = > *9 h*. We calculated a weighted day average score ([5*weekday + 2*weekend days]/7) with higher scores reflecting more time spend with hedonic activities.

**Trait hedonic capacity.** Trait hedonic capacity was assessed with the Trait Hedonic Capacity Scale (Bernecker & Becker, 2021). The scale was validated in a series of laboratory and field studies and consists of 10 items measuring *hedonic success* (e.g., “I am good at pursuing my desires”) and the experience of *intrusive thoughts* (e.g., “I often think about my duties even while I am enjoying a good moment”, recoded). Items were rated on a 5-point scale from 1 = *not at all* to 5 = *very much* (Cronbach’s α = 0.89). Higher scores reflect higher trait hedonic capacity.

## Results

**Table 1:** *Means, Standard Deviations, and Zero-Order Correlations with Confidence Intervals for Study 1*

Variable	*M*	*SD*	1	2	3
1. Trait Hedonic Capacity	3.25	0.75			
2. Hedonic Quantity	2.22	1.55	.26**		
			[.14, .36]		
3. WHO-5 Well-Being	3.90	0.84	.40**	.19**	
			[.30, .49]	[.08, .30]	
4. Physical Symptoms	1.57	0.43	− .47**	− .15*	− .61**
			[-.55, − .37]	[-.26, − .04]	[-.67, − .53]

*Note. M* and *SD* are used to represent mean and standard deviation, respectively. Values in square brackets indicate the 95% confidence interval for each correlation. The confidence interval is a plausible range of population correlations that could have caused the sample correlation (Cumming, 2014). * indicates *p* < .05. ** indicates *p* < .01

Zero-order correlations show a medium-sized positive relationship between trait hedonic capacity and time spent with hedonic activities (see Table 1). People with higher trait hedonic capacity spent more time with hedonic activities with a medium effect size (Cohen, 1992). The correlation between trait hedonic capacity and hedonic quantity was similar for weekdays, *r*(291) = 0.22, *p* < .001, and weekend days, *r*(291) = 0.24, *p* < .001. Further, replicating previous findings, trait hedonic capacity was positively related to general well-being and negatively related to physical symptoms for anxiety, depression, and somatization. Both effects were medium-to-large in size.

Next, we ran two step-wise multiple regression models to test whether spending more time with hedonic activities accounted for the relationships between trait hedonic capacity and general well-being/physical symptoms (see Table 2). In step 1, we predicted both outcomes with trait hedonic capacity and controlled for gender due to gender differences in trait hedonic capacity reported previously (Bernecker & Becker, 2021). In step 2, we added hedonic quantity. Time spent with hedonic activities was a positive predictor for general well-being and a negative predictor of physical symptoms. However, the effects of trait hedonic capacity on general well-being/physical symptoms remained significant. Additionally, we ran two causal mediation analyses (Imai et al., 2010) in the mediate package (Tingley et al., 2014), which confirmed that the indirect effect was not significant for life satisfaction, *b* = 0.05, 95% CI [-0.01, 0.15], *p =* .110, nor for physical symptoms, *b* = -0.007, 95% CI [-0.04, 0.02], *p =* .630. Even though spending more time with hedonic activities is positively related to well-being it does not seem to explain *why* people higher in trait hedonic capacity experience greater well-being and less physical symptoms.

## Brief discussion

Study 1 suggests that people with higher trait hedonic capacity spent more time with hedonic activities and experience greater well-being. Although time spent with hedonic activities is positively associated with well-being, it does not account for the positive effects trait hedonic capacity on well-being. One major limitation of this study is that the data was collected in April 2021 during lockdown due to the COVID-19 pandemic. Results might therefore not generalize to non-lockdown times. A second limitation is the self-report of time spent with hedonic activities which might be inaccurate. To address both limitations, we re-analyzed an experience-sampling data set that was collected before the COVID-19 pandemic.

**Table 2:** *Multiple Regression Models Predicting Well-Being by Trait Hedonic Capacity and Hedonic Quantity*

		General Well-Being							Physical Symptoms						
		*b*	*95% CI*		*SE*	*t*	β	*p*	*b*	*95% CI*		*SE*	*t*	β	*p*
Step 1															
	Intercept	4.07	3.86	4.28	0.11	37.95			1.49	1.39	1.59	0.05	28.38		
	Male vs. Female	-0.21	-0.44	0.03	0.12	-1.76	− .10	.080	0.10	-0.02	0.21	0.06	1.66	.09	.097
	Male vs. Diverse	0.07	-1.03	1.17	0.56	0.12	.01	.905	-0.05	-0.59	0.48	0.27	-0.19	− .01	.846
	Trait Hedonic Capacity	0.32	0.23	0.41	0.05	7.11	.38	< .001	-0.19	-0.24	-0.15	0.02	-8.78	− .45	<.001
Step 2															
	Intercept	4.06	3.85	4.28	0.11	37.78			1.49	1.39	1.59	0.05	29.14		
	Male vs. Female	-0.20	-0.44	0.03	0.12	-1.71	− .09	.089	0.09	-0.02	0.20	0.06	1.61	.08	.108
	Male vs. Diverse	0.00	-1.08	1.08	0.55	-0.01	.00	.995	-0.01	-0.52	0.50	0.26	-0.04	.00	.970
	Trait Hedonic Capacity	0.29	0.20	0.38	0.05	6.12	.34	< .001	-0.19	-0.23	-0.14	0.02	-8.26	− .44	<.001
	Hedonic Quantity	0.15	0.04	0.26	0.05	2.80	.18	.006	-0.06	-0.11	-0.01	0.03	-2.48	− .15	.014

*Note.* Male vs. Female = dummy coded variable comparing male (0) and female (1) participants. Male vs. Diverse = dummy coded variable comparing male (0) and gender diverse (1) participants

## Study 2

Data for this study was collected in fall 2019 and included an experience-sampling phase as well as a baseline measurement of trait hedonic capacity and a follow-up measurement of well-being. 1 We re-analyzed this data set to replicate the positive relationship between trait hedonic capacity and time spent with hedonic activities. Second, we examined whether hedonic quantity (i.e., time spent) vs. hedonic quality (i.e., enjoyment, relaxation, positive affect) account for the relationship between trait hedonic capacity and well-being. Third, we explore whether and how trait hedonic capacity is related to academic performance based on objective performance data (i.e., grade point average, ECTS points achieved. 2).

## Method

### Study design and procedure

The study consisted of a baseline and follow-up survey and a 7-day experience-sampling phase in between. In this phase, participants received four signals a day randomly distributed between 9 am and 9 pm. Upon receipt of a signal, participants had 30 min to respond to the 2-minute survey. Experience-sampling was done with the P.I.E.L survey app (Jessup et al., 2012) on participants’ private smartphone. The app saves the data locally on the phone which then needed to be sent to the study team via email at the end of the study. Because we were also interested in examining students’ academic performance, only bachelor students of a Swiss university were eligible to participate in the study. In the informed consent, participants agreed to release their official transcript of records to the study team. Out of these records, we examined the number of ECTS points they attempted and achieved in the semester when the study was conducted as well as their grade point average (GPA). Participants were compensated with partial course credit and a bonus payment, if they achieved a 90% response rate during experience-sampling phase (*n* = 74). The study procedure and materials were approved by the institutional review board.

### Participants

A sample of *N* = 224 students (194 female, 30 male, *M*_Age_ = 21.25 years, *SD* = 3.71 years, ranging from 17 to 47 years; all but 2 psychology majors) completed the baseline measure, of which *n* = 180 sent their data file of the experience-sampling phase, which translates into a power of 1 – β = 0.91 to find a small effect in a multiple regression (*f*^2^ = 0.05, α = 0.05). On average participants provided 23 experience samples (range: 4–28).

### Measures

Only the measures relevant for the present research are described. A full list of variables can be found in the stimulus materials on OSF.

### Baseline survey

***Trait hedonic capacity.*** Trait hedonic capacity was assessed with the Trait Hedonic Capacity Scale (Bernecker & Becker, 2021) as described in Study 1 (Cronbach’s α = 0.84).

***Trait self-control.*** We assessed levels of trait self-control with the short-version of the Trait Self-Control Scale (Bertrams & Dickhäuser, 2009; Tangney et al., 2004), that consists of 13 items (e.g., “I am good at resisting temptation”) rated on a 5-point scale from 1 = *not at all* to 5 = *very much* (Cronbach’s α = 0.83).

***Academic performance.*** From participants’ official grade records for the semester when the study was conducted we extracted the ECTS points they achieved. Further, classes were graded either as pass/fail or on a score from 1.00 (worst) to 6.00 (best) in steps of 0.50. We calculated the grade point average (GPA) by weighing each grade with the ECTS points for that class. Because some students only attended classes graded as pass/fail, we only have GPAs for a subsample of *n* = 130 students, which translates into an acceptable power of 1 – β = 0.81 to find a small effect in a multiple regression analysis (*f*^2^ = 0.05, α = 0.05).

### Experience-sampling phase

***Hedonic quantity.*** First, participants named their current activity in an open-ended response and then chose one out of 11 categories (e.g., studies, job, commuting, active leisure, relaxing, other). Then they were asked “Would you classify this activity more as effort/work or pleasure/leisure?” (0 = *effort/work*, 1 = *pleasure/leisure*, adapted from Rom et al., 2020). For each participant, we calculated the proportion of activities classified as pleasure/leisure (*M* = 50%, Range: 13–85%).

***Hedonic quality.*** Hedonic quality was assessed with three indicators. For each signal participants reported their momentary affect on two continuous sliders (0.00 = *very bad* to 1.00 = *very good*; 0.00 = *very tensed* to 1.00 = *very relaxed*) on sliders. Further, they indicated how much they enjoyed the activity on a slider from 0.00 = *not at all* to 1.00 = *very much*. We averaged the three items for each activity (Cronbach’s α = 0.81), but also explored them as separate mediators for the link between trait hedonic capacity and well-being.

### Follow-up survey

**Life satisfaction.** In the follow-up survey, we administered the Life Satisfaction Scale (Diener et al., 1985), which consists of 5 items (e.g., “In most ways my life is close to my ideal”, Cronbach’s α = 0.83) on a 7-point scale from 1 = *strongly disagree* to 7 = *strongly agree*).

## Results

**Preliminary analyses.** Descriptive statistics and zero-order correlations are presented in Table 3. The quantity of hedonic activities was positively correlated with trait hedonic capacity and negatively correlated with trait self-control. Relationships with the three indicators of academic performance were all not significant for trait hedonic capacity and trait self-control. The quantity of hedonic activities was negatively correlated with ECTS points and GPA but the size of the effect was small and not significant.

**Table 3:** *Means, Standard Deviations, and Zero-Order Correlations with Confidence Intervals for Study 2*

Variable	*M*	*SD*	1	2	3	4	5	6
1. Trait Hedonic Capacity	3.06	0.65						
2. Trait Self-Control	3.10	0.63	.12					
			[-.03, .26]					
3. Hedonic Quantity ESM	0.50	0.13	.24**	− .17*				
			[.09, .37]	[-.31, − .02]				
4. Hedonic Quality ESM	0.61	0.10	.45**	.21**	.37**			
			[.33, .56]	[.07, .35]	[.23, 0.49]			
5. Life Satisfaction	5.01	1.18	.27**	.16*	− .00	.27**		
			[.12, .40]	[.01, .30]	[-.15, .15]	[.12, .40]		
6. ECTS Points	10.84	7.74	− .12	.10	− .19*	− .09	.22**	
			[-.26, .03]	[-.04, .25]	[-.33, − .05]	[-.23, .06]	[.08, .36]	
7. GPA	4.98	0.46	.03	.09	− .15	.02	.09	.03
			[-.16, .22]	[-.10, .28]	[-.33, .05]	[-.18, .21]	[-.11, .28]	[-.17, .22]

*Note.* ESM = experience-sampling method. *M* and *SD* are used to represent mean and standard deviation, respectively. Values in square brackets indicate the 95% confidence interval for each correlation. The confidence interval is a plausible range of population correlations that could have caused the sample correlation (Cumming, 2014). * indicates *p* < .05. ** indicates *p* < .01

**Hedonic quantity**. To test the incremental relationship between trait hedonic capacity and hedonic quantity, we ran a multiple regression model controlling for gender, trait self-control, and the number of experience samples. Results showed that gender was not related to hedonic quantity, β = 0.00, *b* = 0.00, 95% CI [-0.06, 0.06], *SE =* 0.03, *t*(175) = 0.00, *p =* .997, while the number of experience samples was positively related to it, β = 0.16, *b* = 0.01, 95% CI [0.00, 0.01], *SE =* 0.002, *t*(175) = 2.21, *p =* .028. People with more experience samples were more likely to be engaged in hedonic goal pursuit. Replicating the results of Study 1, trait hedonic capacity was a significant positive predictor of hedonic quantity, β = 0.27, *b* = 0.04, 95% CI [0.02, 0.05], *SE =* 0.01, *t*(175) = 3.66, *p* < .001. Interpreting the unstandardized *b*s as effect size, the results suggest that people higher (+ 1 SD) vs. lower (− 1 SD) in trait hedonic capacity reported in 8% more signals to be involved in a hedonic activity. Trait self-control was a significant negative predictor of it, β = − 0.23, *b* = -0.03, 95% CI [-0.05, − 0.01], *SE =* 0.01, *t*(175) = -3.17, *p =* .002, with a 6% difference in the proportion of signals between people high and low in trait self-control.

**Hedonic quality.** We ran the same models to predict hedonic quality. Results showed that gender and number of samples were unrelated to hedonic quality, *t*s < 1. Trait hedonic capacity was a positive predictor, β = 0.43, *b* = 0.04, 95% CI [0.03, 0.05], *SE =* 0.01, *t*(175) = 6.31, *p <* .001, Δ*R*^2^ = 0.17, with a medium-to-large effect size. Trait self-control was also a positive predictor but with a smaller effect size, β = 0.16, *b* = 0.02, 95% CI [0.00, 0.03], *SE =* 0.01, *t*(175) = 2.31, *p =* .022, Δ*R*^2^ = 0.02. 3

**Life satisfaction.** Next, we aimed to test whether hedonic quantity or hedonic quality work as mediators for the relationship between trait hedonic capacity and life satisfaction. We regressed trait hedonic capacity on life satisfaction, controlling for gender and trait self-control. We replicated the positive relationship between trait hedonic capacity and life satisfaction, β = 0.26, *b* = 0.30, 95% CI [0.13, 0.47], *SE =* 0.09, *t*(172) = 3.54, *p <* .001. Next, we added hedonic quantity to this model and found that it was unrelated to life satisfaction, *t* < 1. Causal mediation analyses confirmed that the indirect effect between trait hedonic capacity, hedonic quantity, and life satisfaction was not significant either, *b* = -0.03, 95% CI [-0.15, 0.08], *p =* .582, while the direct effect of trait hedonic capacity on life satisfaction remained significant, *b* = 0.62, 95% CI [0.28, 0.96], *p <* .001. Replicating the findings of Study 1, hedonic quantity does not seem to account for the positive relationship between trait hedonic capacity and well-being (i.e., life satisfaction).

Next, we added hedonic quality instead of hedonic quantity to the model and found that it was positively related to life satisfaction, β = 0.16, *b* = 1.97, 95% [0.00, 3.94], *SE =* 1.00, *t*(171) = 1.98, *p =* .050. Causal mediation analyses showed a marginally significant indirect effect, *b* = 0.16, 95% CI [-0.01, 0.38], *p =* .076, while the direct effect remained significant, *b* = 0.53, 95% CI [0.19, 0.88], *p =* .004. When we tested the three indicators of hedonic quality separately, the indirect effect was significant for affect valence, *b* = 0.19, 95% [0.04, 0.38], *p =* .014, not significant for affect arousal, *b* = 0.14, 95% CI [-0.05, 0.35], *p =* .156, and not significant for enjoyment, *b* = 0.05, 95% CI [-0.06, 0.18], *p =* .431. Overall, this suggests that especially positive affect experienced during hedonic activities seems to account for some part of the positive relationship between trait hedonic capacity and life satisfaction.

**Academic performance.** Last, we ran multiple regression models to test whether trait hedonic capacity was related to academic performance (i.e., ECTS points, GPA), when controlling for trait self-control and gender. We found that trait hedonic capacity was unrelated to ECTS points, β = − 0.06, *b* = -0.44, 95% CI [-1.47, 0.59], *SE =* 0.52, *t*(218) = -0.84, *p =* .403, and GPA, β = − 0.01, *b* = -0.00, 95% CI [-0.09, 0.08], *SE =* 0.04, *t*(126) = -0.11, *p =* .914. But also trait self-control was unrelated to both ECTS points, β = 0.08, *b* = 0.60, 95% CI [-0.43, 1.62], *SE =* 0.52, *t*(218) = 1.15, *p =* .253, and GPA, β = 0.14, *b* = 0.06, 95% CI [-0.02, 0.14], *SE =* 0.04, *t*(126) = 1.60, *p =* .112.

## Brief discussion

The results of Study 2 replicate and extend the findings of Study 1: Students higher in trait hedonic capacity engaged more often in hedonic activities but this difference cannot explain why they experience greater well-being. Rather, hedonic quality and specifically positive affect experienced during hedonic activities seems to account at least for some part of the relationship. Further, trait hedonic capacity was not negatively related to student’s objective academic performance. Despite spending more time with hedonic activities, students with higher trait hedonic capacity reached a similar amount of ECTS points and GPA. One limitation of this study is the homogenous student sample, which we aimed to address in Studies 3 and 4.

## Study 3

Building upon the finding of Study 2 that trait hedonic capacity is unrelated to academic performance, this study aimed to examine the relationship between trait hedonic capacity and job performance in two diverse adult samples. As indicator for job performance, we assessed level of income and controlled for gender, highest level of education, and work hours as potential confounds.

## Method

### Participants

We recruited two diverse adult samples online. Sample A consists of *N* = 165 German-speaking adults (119 females, 44 males, 2 NA, *M*_age_ = 35.30, *SD*_age_ = 13.72, range: 18–61) that were recruited on online social networks (e.g., Facebook, LinkedIn) for a 10-min online questionnaire on well-being in the workplace. In this sample, 57.6% worked full-time, 40.0% worked part-time, 2.3% studied or were retired. Sample B consists of *N* = 350 English-speaking adults (213 females, 136 males, 1 diverse; *M*_age_ = 35.30, *SD*_age_ = 11.30, range: 18–77) recruited on prolific.com residing in the UK and US. Participants received £5.00 as compensation for completion of a 40-min online questionnaire. In Sample B, 50.0% worked full-time, 21.3% worked part-time, 10.9% were looking for work, 3.7% were retired, and 14.1% studied.^4^ In both samples, we had sufficient power to find small effect in a multiple regression analysis (*f*^2^ = 0.05, α = 0.05), 1 – β_Sample A/B_ = 0.81/0.98.

### Measures

Measures were presented in the following order.

**Education.** We asked participants in both samples to indicate their highest level of education on a 7–point scale adapted to the German school system with 1 = Less than secondary school, 2 = Lower secondary school, 3 = Higher secondary school, 4 = A-level/university certificate, 5 = Undergraduate degree, 6 = Postgraduate degree, 7 = Doctoral degree. In Sample B participants answered the same question 7-point scale adapted to the UK school system from 1 = Less than GCSE/ Middle school, 2 = GCSE/ Middle school graduate, 3 = A-Level/ high school graduate, 4 = Undergraduate degree (Bachelor), 5 = Postgraduate degree (Master), 6 = Professional degree, and 7 = Doctorate.

**Income.** Further, participants in Sample A indicated their monthly income (after taxes) on a 10-point scale from 1 = below 500 €, 2 = 1000 € to less than 1500 €, 3 = 1500€ to less than 2000 €,…, 9 = 4000€ to less than 5000 €, 10 = above 5000 €. Participants in Sample B indicated their yearly household income (before taxes) in the currency they indicated beforehand (i.e., GBP, USD, €) on a 13-point scale from 1 = less than 10’000, 2 = 10’000–15’999, 3 = 16’000–19’999, 4 = 20’000–29’999, 5 = 30’000–39’999, …, 11 = 90’000–99’999, 12 = 100’000-149’999, 13 = More than 150’000.

**Work status.** In both samples, we asked participants to indicate their work status which they indicated as being 1 = working full time, 2 = working part-time, 3 = looking for work, 4 = retired, 5 = full time student, 6 = part time student.

**Work hours.** In Sample A, we additionally asked participants to indicate the number of work hours in a week in an open response field. We had 27 missings on this variable (e.g., people indicating that they studied, were retired, or looking for a job).

**Trait hedonic capacity and trait self-control.** We used the same measures as described previously to assess trait hedonic capacity (Cronbach’s α = 0.87/0.84) and trait self-control (Cronbach’s α = 0.77/0.86).

## Results

**Preliminary analyses.** In both samples, trait hedonic capacity was positively and significantly correlated with income (see Table 4). However, trait hedonic capacity was also positively correlated with trait self-control and work hours as potential confounds. The correlation with highest level of education was close to zero in both samples and not significant.

**Table 4:** *Means, Standard Deviations, and Zero-Order Correlations with Confidence Intervals for Study 3*

Variable	*M*	*SD*	1	2	3	4
Sample A						
1. Trait Hedonic Capacity	3.15	0.71				
2. Trait Self-Control	3.51	0.64	.17*			
			[.01, .31]			
3. Education^a^	-	-	− .03	− .00		
			[-.12, .06]	[-.09, .09]		
4. Income	4.01	2.71	.20*	.04	.23**	
			[.05, .35]	[-.12, .20]	[.14, .30]	
5. Work hours	30.10	14.38	.12	.10	.15**	.72**
			[-.05, .28]	[-.07, .26]	[.06, .23]	[.63, .80]
Sample B						
1. Trait Hedonic Capacity	2.87	0.69				
2. Trait Self-Control	2.90	0.73	.24**			
			[.14, .33]			
3. Education^a^	-	-	.06*	.02		
			[.01, .11]	[-.03, .08]		
4. Income	5.98	3.62	.13*	.08	.18**	
			[.02, .23]	[-.03, .18]	[.14, .22]	

*Note.*^a^ Estimates for education represent Kendall’s rank correlation τ. *M* and *SD* are used to represent mean and standard deviation, respectively. Values in square brackets indicate the 95% confidence interval for each correlation. The confidence interval is a plausible range of population correlations that could have caused the sample correlation (Cumming, 2014). * indicates *p* < .05. ** indicates *p* < .01

**Income.** In both samples, we ran a multiple regression model predicting income by trait hedonic capacity and controlled for gender, highest level of education, work hours (in Sample A)/ work status (in Sample B), and trait self-control as potential third variables. In Sample A, gender had a negative association with income, β = − 0.26, *b* = -1.43, 95% CI [-2.09, -0.79], *SE* = 0.34, *t*(114) = -4.25, *p* < .001, indicating that women earned less than men. Work hours were positively correlated with income, β = 0.52, *b* = 1.25, 95% CI [0.94, 1.57], *SE* = 0.16, *t*(114) = 7.81, *p* < .001. Highest level of education was entered as dummy variables with secondary school being the omitted group. This group only differed significantly from people with undergraduate studies, β = 0.28, *b* = 1.69, 95% CI [0.13, 3.24], *SE* = 0.78, *t*(114) = 2.15, *p* = .034. The other comparisons were not significant, *t*s < 1.88. Trait self-control was positively associated with income and the effect was marginally significant, β = 0.11, *b* = 0.28, 95% CI [-0.00, 0.57], *SE* = 0.14, *t*(114) = 1.97, *p* = .051. Importantly, trait hedonic capacity was not significantly associated with income when controlling for these variables, β = 0.06, *b* = 0.15, 95% CI [-0.14, 0.44], *SE* = 0.15, *t*(114) = 1.01, *p* = .314.

In Sample B, gender was not significantly associated with income, β = − 0.02, *b* = -0.15, 95% CI [-0.98, 0.67], *SE* = 0.42, *t*(237) = -0.37, *p* = .714. Work status was significantly associated with income with people working part-time earning less, β = − 0.14, *b* = -0.98, 95% CI [-1.83, -0.13], *SE* = 0.42, *t*(237) = -2.26, *p* = .025. Trait self-control was not significantly associated with income, β = 0.08, *b* = 0.28, 95% CI [-0.13, 0.68], *SE* = 0.21, *t*(237) = 1.33, *p* = .184. Trait hedonic capacity was positively associated and the effect marginally significant, β = 0.11, *b* = 0.36, 95% CI [-0.05, 0.76], *SE* = 0.21, *t*(237) = 1.75, *p* = .082.

## Brief discussion

In two diverse adult samples, trait hedonic capacity was positively rather than negatively associated with income. However, the relationships were small in both samples and dropped to non-significance in Sample A and marginal significance in Sample B when we controlled for third variables (i.e., gender, education, work hours). Trait self-control was positively associated with income but the effect was small and only marginally significant in Sample A and non-significant in Sample B. Overall, the relationships for both traits were rather small and should be interpreted with caution. Further, a major limitation of this study is that we assessed job performance only by people’s income as a single indicator.

## Study 4

The aim of this study was to address this limitation of Study 3 using several indicators of job performance. Five indicators of job performance are commonly used in the literature: income, hierarchy level, promotions, absenteeism, and presenteeism (Abele et al., 2011; Dette et al., 2004; Kessler et al., 2003). Absenteeism is conceptualized as hours absent from work (e.g., because of illness, coming in late) and presenteeism as actual performance in relation to possible performance (Kessler et al., 2003). The study was pre-registered on aspredicted.org: https://aspredicted.org/YL8_PD5.^5^ Based on the findings of Study 3, we pre-registered a small positive relationship for both trait hedonic capacity and trait self-control with job performance.

## Method

### Participants

The sample consisted of *N* = 268 participants (176 women, 92 men, *M*_age_ = 30.44, *SD*_age_ = 9.18, range: 18–65 years) that we recruited on online social networks (e.g., Facebook, LinkedIn). Inclusion criteria were being 18 or older and employed at least 50% of the time (equivalent to at least 17 working hours per week). Participants indicated to work on average 40.02 h per week (*SD* = 7.90, range: 18–70 h). Again, this study was sufficiently powered to find a small effect in a multiple regression analysis (*f*^2^ = 0.05, α = 0.05), 1 – β = 0.98.

### Measures

**Job performance.** Income was assessed on an 10-point Likert-type scale from 1 = < *500* Euro to 11 = > *5000 Euro*. Hierarchical level was assessed with 3 items (adapted from Abele et al., 2011, e.g., “Do you have authority to delegate work?”, 1 = *yes*, 0 = *no*). Items were averaged to one indicator of hierarchy level. Job promotion was assessed with 2 items (adapted from Dette et al., 2004; Greenhaus, et al., 1990, i.e., “Did you ever get promoted in your job?” 1 = *yes*, 0 = *no*, “If yes, how often did you get promoted in your job up until now?” *open response*). The number of promotions was used as indicator of performance. Absenteeism was assessed as the difference between hours of work reported for the past 7 days and hours of work expected from the employee for the same time period (adapted from Kessler et al., 2003). Presentism was assessed with 2 items (adapted from Kessler et al., 2003): One item asking participants to judge the performance of the average employee on their job and the second to judge their own job performance in the past 4 weeks (0 = *worst performance*, 10 = *best performance*). We calculated the ratio of own and average employee performance as indicator of presentism. Because indicators were measured on different response scales, we z-transformed all of them before we averaging to one indicator of job performance. We replicated our analyses with structural equation modeling and building a latent job performance indicator.

**Trait hedonic capacity and trait self-control.** We used the same measures as described previously to assess trait hedonic capacity (Cronbach’s α = 0.87) and trait self-control (Cronbach’s α = 0.82).

## Results

**Preliminary analyses.** Table 5 summarizes the descriptive statistics and zero-order correlations. Trait hedonic capacity was again positively correlated with income, however, the effect was smaller and only marginally significant in this study. Trait hedonic capacity was largely uncorrelated with the other indicators of job performance. Trait self-control was positively and significantly associated with presentism, marginally significantly with promotions and uncorrelated with the remaining three job performance indicators.

**Table 5:** *Means, Standard Deviations, and Zero-Order Correlations with Confidence Intervals for Study 4*

Variable	*M*	*SD*	1	2	3	4	5	6	7
1. Trait Hedonic Capacity	3.28	0.71							
2. Trait Self-Control	3.32	0.61	.10						
			[-.02, .22]						
3. Income	5.11	2.27	.12	.07					
			[-.01, .23]	[-.05, .19]					
4. Hierarchy	0.43	0.33	− .07	.04	.32**				
			[-.18, .05]	[-.08, .15]	[.21, .43]				
5. Promotion	0.74	1.18	− .10	.11	.39**	.45**			
			[-.23, .04]	[-.03, .24]	[.27, .49]	[.33, .55]			
6. Absenteeism	-8.15	47.27	.04	− .07	− .20**	− .09	− .15*		
			[-.08, .16]	[-.19, .05]	[-.31, − .08]	[-.21, .03]	[-.28, −.02]		
7. Presenteeism	1.12	0.27	− .07	.18**	.04	.15*	.11	−.04	
			[-.19, .05]	[.06, .29]	[-.08, .16]	[.03, .26]	[-.03, .24]	[-.16, .08]	
8. Work Hours	40.02	7.90	− .05	.02	.38**	.27**	.15*	−.09	.00
			[-.17, .07]	[-.10, .14]	[.28, .48]	[.15, .37]	[.02, .28]	[-.21, .03]	[-.12, .12]

*Note. M* and *SD* are used to represent mean and standard deviation, respectively. Values in square brackets indicate the 95% confidence interval for each correlation. The confidence interval is a plausible range of population correlations that could have caused the sample correlation (Cumming, 2014). * indicates *p* < .05. ** indicates *p* < .01

**Job performance.** Because the five indicators income, hierarchy, promotion, absenteeism, and presentism represent quite different aspects of job performance, we used structural equation modeling to estimate a latent job performance variable, based on these five indicators. For that, we used AMOS version 27 and first constructed a measurement model. In AMOS, only complete data sets can be used for this (*n* = 260, *M*_*age*_ = 30.46, *SD*_*age*_ = 9.20, range: 18–65 years). Promotion was selected as the reference indicator and its regression weight was fixed to 1. The model fit measures as well as the descriptive quality criteria indicate a good fit of the measurement model to the data, χ^2^(5) = 5.02, *p* = .413, χ^2^/*df* = 1.005, RMSEA = 0.004, SRMR = 0.030, GFI = 0.992, AGFI = 0.976, NFI = 0.950, CFI = 1.000. Next, we estimated a path model and predicted the latent job performance variable by trait hedonic capacity and trait self-control and controlled for gender and work hours. Trait hedonic capacity was not related to job performance, β = 0.01, *b* = 0.002, *SE* = 0.09, *t*(30) = 0.02, *p* = .983. The effect of trait self-control was positive but small and only marginally significant, β = 0.12, *b* = 0.19, *SE* = 0.11, *t*(30) = 1.67, *p* = .094.

## Brief discussion

In this study, trait hedonic capacity was positively correlated with income but unrelated to other indicators of job performance (e.g., promotions, presenteeism). Overall people higher in trait hedonic capacity do not perform better nor worse in their jobs than people lower in trait hedonic capacity. Trait self-control was positively associated with presenteeism but not significantly related to the other job performance indicators.

## General discussion

In past decades, self-control research has demonized people’s hedonic goals as potential threats to their presumably more important long-term goals and related outcomes. As a result, we do not know much about the actual consequences of hedonic goal pursuit for important outcomes such as well-being or performance. The present research examined whether people’s capacity to pursue hedonic goals successfully (i.e., trait hedonic capacity) is related to how much time people spend with hedonic activities (i.e., hedonic quantity) and explored possible negative consequences of trait hedonic capacity for academic and job performance. The findings of Study 1 and 2 suggest that people with higher trait hedonic capacity indeed spend more time with hedonic activities. However, spending more time with hedonic activities did not explain the positive relationship between trait hedonic capacity and well-being (e.g., life satisfaction). Rather, hedonic quality and especially positive affect elicited by these activities seems to account at least for parts of the relationship between trait hedonic capacity and well-being. Further, we did not find support for the idea that higher levels of trait hedonic capacity might come at a cost to people’s academic (Study 2) or job performance (Study 3 and 4). Further, across three adult samples (Study 3 and 4) we found only weak evidence for a positive relationship between trait self-control and job performance.

### Trait hedonic capacity, hedonic quantity, and well-being

Previous research showed that being able to pursue hedonic goals successfully is positively related to people’s well-being and as such an important aspect of adaptive self-regulation (Bernecker & Becker, 2021). An open question that remained from this research was *why* trait hedonic capacity is positively related to well-being. Field studies demonstrated that trait hedonic capacity predicts hedonic quality in everyday life, that is, more positive affect, relaxation, and enjoyment during hedonic goal pursuit. However, this research did not test whether the quality of hedonic experiences drives the positive effects of trait hedonic capacity on well-being. Replicating previous research (Hofmann et al., 2014), we found that positive affect in everyday life was a significant predictor of people’s well-being (i.e., life satisfaction). However, a substantial part of the positive relationship remained when controlling for hedonic quality, suggesting that experiencing more positive affect in everyday life is only part of the mechanism.

Hedonic quantity was positively related to well-being in Study 1 but unrelated to well-being in Study 2. Given this inconsistency, we cannot ultimately conclude whether spending more time with hedonic goal pursuit is conducive or unrelated to well-being, and remains an open question for future research. The assessment of hedonic quantity is not straightforward and might have caused some of the inconsistency. Research suggests that people might have a hard time judging the time they spend on activities especially if they are fun (e.g., Droit-Volet & Meck 2007; Gable & Poole, 2012). Given that people high in trait hedonic capacity are more likely to have experienced pleasurable affective states during hedonic activities, they might have underreported time they spent with it in Study 1. Speaking against that argument, Study 2 replicated the positive association between trait hedonic capacity and hedonic quantity using the experience-sampling method which is well-suited for studying how people spend their time (Mehl & Conner, 2012; Taquet et al., 2016). Another difficulty in comparing the results of Study 1 and Study 2, are the different measures of well-being that were used. Study 1 measured general well-being and physical symptoms, whereas Study 2 utilized a measure of life satisfaction. More research is needed to draw final conclusions about the relationship between the time people spent with hedonic activities and well-being.

Another open question that remains regarding hedonic quantity is whether people higher in trait hedonic capacity initiate hedonic goal pursuit more often and/or are more persistent once they started, which is related to recent theoretical considerations regarding initiatory/start and inhibitory/stop self-control (Converse et al., 2014; Hoyle & Davisson, 2016). It seems plausible that persistence in hedonic goal pursuit should be positively related to hedonic quality. If people experience more positive affective states during hedonic activities, which people with higher trait hedonic capacity typically do, they might be motivated to prolong the activity speaking for greater persistence. Likewise, from a reinforcement perspective, hedonic quality might increase the likelihood of initiating hedonic goal pursuit in the future. If people are more successful in pursuing their hedonic goals they might also turn to these goals more often. In order to address this question future research should assess people’s choices of investing time into hedonic versus long-term goal pursuit as well as their persistence in it.

### Trait hedonic capacity and performance

Besides extending knowledge on the mechanisms driving positive effects of trait hedonic capacity on well-being, we did not find indication that these effects might come at a cost of people’s academic or job performance. If anything, we found small positive correlations between trait hedonic capacity and income (Study 3 and 4). Because these studies were correlational, we cannot rule out that the positive association is based on confounds (e.g., socio-economic status) and can also not infer the direction of causality. It may be that people who have more financial means are more likely to have the capability to “relax well” and “do what I feel like doing”. This is mainly because many pastimes and leisure activities are considered luxuries that come with having a reliable job and other forms of privilege. However, Study 2 was longitudinal and yielded objective data on students’ academic performance. Unfortunately, we did not assess trait hedonic capacity after students’ exams, such that we were not able to run cross-lagged analyses. Taken together, the correlational data of three studies suggest that trait hedonic capacity is largely unrelated to academic and job performance. The positive effects of high trait hedonic capacity do not seem to come at a cost to people’s performance, despite these people spending more time with hedonic activities which was negatively related to academic performance in Study 2. Hedonic quantity does seem to matter for (academic) performance, however, there might be compensatory mechanisms at work. For instance, research suggests that successful recovery after work and on the weekend is a positive predictor of people’s job performance (Binnewies et al., 2009, 2010).

Another important point to discuss is that we overall found weak evidence for the positive relationship between trait self-control and people’s academic and job performance. Similar to previous studies, we used both objective and self-report measures of academic and job performance and administered a standard measure of trait self-control (Tangney et al., 2004). As mentioned in the introduction the numbers of studies investigating the link between self-control and performance is still quite small with the majority looking at academic performance (de Ridder et al., 2012). The present research therefore adds to the existing literature enabling future meta-analyses to derive at a reliable mean effect size for the self-control-performance-link.

### Strength, limitations, and constraints to generalizability

In this research, we combined self-report with objective measures, cross-sectional with longitudinal/extensive measurement designs and investigated student as well as diverse adult samples. Further, we replicate our core findings across multiple studies. However, there are also limitations of the present work that we would like to discuss. One limitation that we discussed earlier is the conceptualization and measurement of hedonic quantity and the necessity to disentangle initiation and persistence of hedonic goal pursuit in future studies. Further, with regard to job performance, future research should consider more objective measures such as ratings by managers or team members or looking into jobs that provide objective performance data (e.g., sales). Last, all of our studies have a correlational design, which limits the conclusion that we can draw from the mediation analyses (Fiedler et al., 2011). In the future, experimental studies (e.g., interventions) could test causal effects of increasing hedonic quality and/or hedonic quantity experimentally on outcomes such as well-being or performance. The present study lays the foundation for such experimental studies by showing that adverse effects of such interventions are unlikely.

As suggested by (Simons et al., 2017) we would like to discuss constraints to generalizability of our findings. First, all of our samples were drawn from Western countries (i. e., Germany, Switzerland, UK, US), and might therefore not generalize to other countries or societies with different values and socio-economic systems. Alongside with the focus on samples from Western societies, our findings are bound to their current historical or temporal context in which hedonic goals and their pursuit are viewed negatively and subordinate to long-term goal pursuit. However, puritanical norms and the moralization of harmless hedonic behaviors is not a modern phenomenon and one that spans across the globe (Fitouchi et al., 2022). We would expect to find similar results in other societies with relatively strong puritanical norms.

## Conclusion

The present research suggests that hedonic quality, especially positive affect experienced during hedonic goal pursuit, might be part of the reason why people high in trait hedonic capacity experience greater well-being. Further, despite spending more time with hedonic goal pursuit in their everyday life, we did not find any significant negative associations between trait hedonic capacity and academic or job performance. Open questions remain regarding initiation and persistence in hedonic goal pursuit as possible reasons for higher hedonic quantity.

## Data Availability

All study materials, data, and code are publicly available on the Open Science Framework: https://osf.io/fvjez/?view_only=b57f25deb1dd4d2db019f54f8b4667b4.

## Footnotes

1 Data of this study have been reported in Bernecker & Becker (2021) but with a focus on a different research question and reporting different relationships than the one reported here.

2 ECTS (European Credit Transfer and Accumulation System) points are a grading system used by many universities across Europe to measure the workload and performance of their students. Each ECTS credit is equivalent to 25-30 hours of student workload, including attendance at lectures, tutorials, practical work, as well as time spent writing essays, completing assignments and preparing for exams.

3 Results for the separate indicators of hedonic quality are reported in Bernecker & Becker (2021) and will therefore not be repeated here to avoid double-reporting.

4 Parts of the data collected in this study were reported in Bernecker & Becker (2021) with a focus on the association between trait hedonic capacity and well-being.

5 The pre-registration also includes hypotheses about hedonic behaviors at work and their relationship to job performance. As these relationships are not directly related to the present research question they are not reported in this publication.
